# Senior executive characteristics: Impact on ESG practices and corporate valuation relationship

**DOI:** 10.1371/journal.pone.0303081

**Published:** 2024-07-11

**Authors:** Chunhua Cai, Yannan Geng, Fufei Yang

**Affiliations:** Economics and Management School, Wuhan University, Wuhan, Hubei, China; University of Malta, MALTA

## Abstract

In recent years, sustainable development and green growth and performance of companies in environmental, social, and corporate governance (ESG) has received widespread attention from all sectors of society. Based on panel data of A-share listed companies in China from 2009 to 2022, this study employs a two-way fixed effects model to explore the mechanism of the relationship between ESG practices and corporate value, as well as the moderating effect of executive characteristics within this relationship. The results indicate a robust positive relationship between ESG practices and corporate value. ‎However, this relationship is moderated by the academic backgrounds of senior ‎executives, who negatively influence it, and by male executives, who exert a positive ‎moderating effect. Furthermore, this study reveals the variable impacts of ESG practices in different corporate ‎settings, industries, and institutional frameworks. Moreover, it demonstrates how ESG practices ‎boost corporate value through an enhanced reputation and increased government innovation ‎subsidies. It offers new insights on the strategic value of ESG for corporations and ‎policymakers. It also extends the theoretical framework by integrating attention-based and upper ‎echelons perspectives into the ESG discourse. ‎

## Introduction

In contrast to Friedman’s classic assertion that “The social responsibility of business is to increase its profits,” Nobel laureate Jean Tirole provides a counterargument emphasizing the congruence between the objectives of economic agents and broader societal good, advocating a concerted effort to foster societal well-being. In this context, sustainable development and green initiatives have become the highlight of corporate strategy, with environmental, social, and corporate governance (ESG) performance being a focal point across all sectors of society. Initially introduced by the United Nations Environment Program in 2004, ESG represents an investment philosophy that evaluates an enterprise’s environmental, social, and governance performance. As a progressive iteration of corporate social responsibility [1], influenced by external factors, ESG aligns with the multifaceted concept of “innovation, coordination, green, openness, and sharing,” providing a quantifiable framework for sustainable development. Recent scandals have prompted corporate introspection into the equilibrium between business interests and societal responsibilities, emphasizing ESG practices as a means of enhancing social value while achieving business value.

With the growing acceptance of ESG, scholars investigate its essence, evolution, and effect on corporate growth [2, 3] and focus on financial performance [4, 5]. The prevailing literature is segmented into four viewpoints regarding the ESG–corporate value correlation: positive, negative, non-linear, and indirect. Most suggest a positive relationship, with ESG engagement enhancing corporate value through various channels such as reduced financial constraints, operational efficiency, and risk mitigation [6–8]. Some argue for a negative [9, 10] or non-linear connection [11, 12] while some propose indirect associations [13, 14].

The predominant view suggests a positive relationship between ESG practices and corporate value, mediated by factors such as alleviating financing constraints [15], enhancing operational efficiency [16], and mitigating financial risks [17]. The aforementioned literature provides useful references for understanding the relationship between ESG practices and corporate value and the channels of interaction between them. However, studies have not reached a consensus on the relationship between ESG practices and corporate value. Moreover, as the outcome of collective strategic decision-making by corporate executives, ESG practices have a greater impact on strategic decisions than external environments, industries, sizes, and operational decision-making characteristics. The influence of executive characteristics on strategic decisions is more significant; however, current research has not analyzed the impact of executive characteristics on the relationship between ESG and corporate value, indicating a logical gap [18]. Additionally, the transmission mechanism between ESG practices and corporate value remains unclear and requires further investigation.

This study, using Huazheng ESG data for A-share listed enterprises in China from 2009 to 2022, examines how ESG practices influence corporate value and the moderating effects of executive characteristics. The findings validate the positive impact of ESG on corporate value, with additional insights into the moderating roles of executives’ academic backgrounds and gender and the varying influences across ownership types, industry characteristics, and institutional environments. Moreover, this study finds that ESG practices enhance corporate value by improving corporate reputation through government innovation subsidies.

The contributions of this study are threefold. First, it employs a panel data fixed effects model to verify the impact of ESG practices on corporate value. This not only enriches the application field of the attention-based view but also provides theoretical support for how companies can practice sustainable development and social responsibility. Second, it explores the moderating relationship of executive academic background and gender characteristics between corporate ESG and corporate value and analyzes the marginal moderating effects based on quantile regression. This enriches research related to ESG practices and corporate value and expands the application scope of higher order theories, offering new perspectives and considerations for companies on how to formulate ESG strategies based on executive characteristics. Finally, beyond studying the relationship between ESG practices and corporate value, it also analyzes the mechanism of action of the aforementioned relationship through a structural equation model (SEM), which helps us understand how ESG practices impact corporate value, thereby providing more targeted suggestions for companies in formulating related strategies.

The remainder of the study is structured as follows: The following section presents the theoretical analysis and research hypotheses. The next section describes the research design, followed by sections on the empirical results and analysis. The discussion section discusses the results. Finally, the conclusions and implications section concludes the study.

## Theoretical framework and hypotheses development

### ESG practices and corporate valuation

Organizational theory has seen a paradigm shift from an attention-based view to industry-, resource-, and knowledge-based perspectives. This view underscores the critical role of managerial focus in organizational decision-making processes [19]. The attention-based view defines attention as the distribution of time and energy toward specific subjects, issues, opportunities, and threats, as well as particular skills, processes, plans, projects, and programs [20]. The attention allocation method is pivotal in determining the orientation and framework of information processing [21], offering a novel lens for deciphering organizational behavior. The formulation of corporate strategy is influenced by the distribution of attention by decision makers, which affects strategic choices, including investment proposals, and ultimately impacts corporate performance [22]. As the theory matures, based on the attention-based view, research on innovation outcomes [23], technology investments [24], and strategic planning [25] has gradually increased. Ocasio [20] categorizes existing research on the attention-based view into three types: perspective of attention, investment of attention, and selection of attention. The main issue of attention investment research is how organizational decision makers allocate their time and energy to the selection of external environmental stimuli and corresponding strategic responses. For instance, scholars have explored the influence of managerial focus on innovation across five dimensions: past, present, future, external, and internal [23]. They posit that an outward focus by the executive team improves receptivity to innovation, thereby facilitating organizational transformation. Additionally, external focus within executive teams has been linked to improved technical and market outcomes in product innovation [26] and is also considered beneficial for capitalizing on business opportunities and bolstering innovation performance [23].

As the central strategic decision-making unit within a corporation, the executive team must judiciously allocate its limited attention across various facets of organizational development. Attention allocation is a critical determinant of corporate success. The attention of executive teams can be divided into internal and external attention [19, 20]. External attention refers to the allocation of attention by the executive team to the external environment, which encompasses a combination of capabilities and conditions beyond the boundaries of the organization. Drawing on an attention-based framework, this study posits that ESG practices are instrumental in augmenting corporate value. The executive team’s focus on external factors prompts a broader strategic vision [27] that encompasses stakeholder demands beyond shareholder interests, which can garner recognition from diverse stakeholder groups, thereby enhancing performance and corporate value. Furthermore, attention is an essential capability for opportunity identification and creation [28], and the external environment is replete with opportunities to advance corporate performance [29]. Thus, companies can identify and leverage opportunities to boost their performance through an external focus by bolstering environmental protection, social responsibility, and corporate governance. Finally, the executive team’s attention to external factors facilitates the acceptance of new initiatives, thereby positively influencing organizational change [30–32], and consequently enhancing the company’s market value. ESG practices enable corporations to create sustainable external environments, fostering mutual benefits for businesses and society. Consequently, this study proposes the following hypothesis:

**Hypothesis 1 (H**_**1**_**):** ESG practices positively influence corporate value.

### Executive traits as moderators in ESG value creation

Following the 2007 “subprime mortgage crisis” in the US, a shift from the singular focus on “maximizing shareholder value” to a broader ESG perspective was observed in the West. This shift in paradigms effectuated the diversification of ESG practices. Moreover, as an important strategic decision for companies, the value creation effect of ESG practices may be influenced by numerous internal and external factors. Although many studies have explored the external moderating factors that impact the efficacy of ESG practices on corporate value, internal corporate characteristics have received less attention. To address this gap, this study examines the influence of senior executives’ educational backgrounds and gender on the value created by ESG practices, proposing hypotheses grounded in upper echelon theory.

#### Academic backgrounds of senior executives interacting with ESG

The upper echelons theory revolutionized the understanding of senior executive rationality by considering demographics such as age, tenure, and education. It facilitates a more nuanced interpretation of the interplay among executives’ cognitive abilities, insights, values, and strategic decisions [33]. In addition, research indicates that work experience, particularly in academic settings, profoundly influences executive cognition more than other demographic factors [34].

Academic experience significantly shapes personal values, thought processes, and business management approaches. According to the theory, various studies indicate the positive impact of executives’ academic backgrounds on green innovation [35], reduction of audit fees [36], and curtailment of corporate financialization [37]. Conversely, some studies highlight the restrictive influence of academic background on tax avoidance, earnings manipulation [38], and executive indulgence [39, 40], suggesting that academically trained executives are typically more cautious and conservative in their reasoning [41].

Therefore, this study posits that senior executives’ educational backgrounds play a significant moderating role in the relationship between ESG practices and corporate value. Owing to their early academic experiences, corporate executives with academic backgrounds develop a system of self-regulation and supervision [42], which prevents them from exploiting ESG practices to attract capital market attention for a short-term increase in corporate value. However, corporate ESG, as an investment philosophy and a set of corporate evaluation standards focusing on environmental, social responsibility, and governance performance, influences executives with academic backgrounds to approach this trend without hastily allocating resources and without regard for timing, strategy, and efficiency. These executives’ characteristics of rigor, criticality, and self-discipline, formed through academic training [38], prompt them to carefully consider their actions in response to this trend. In summary, owing to the relatively “sober” understanding of ESG practices by academically oriented executives, the impact of ESG practices on corporate value is weakened. Hence, we propose the following hypothesis:

**Hypothesis 2 (H2):** The academic background of senior executives negatively moderates the relationship between ESG practices and corporate value.

#### Gender influence on executive engagement with ESG initiatives

The existence of behavioral differences between genders is almost self-evident. In recent years, research on gender heterogeneity based on the upper echelons theory has gradually increased, and literature from experimental economics and psychology has shown significant differences between genders in aspects such as risk aversion. With the improvement in women’s socioeconomic status and education levels, female members play an increasingly important role in modern corporate management. Existing research indicates that female executives have unique advantages in improving the quality of financial information disclosure [43], reducing corporate agency costs [44], focusing on ethics and morality [45], and reducing opportunism [46]. However, male characteristics also have unique advantages, and in some circumstances, male management is recognized more than female management [47]. Research has underscored the distinct disparities in leadership styles and decision-making approaches between male and female executives. Males generally exhibit a preference for assertive and commanding leadership styles [48], and their decision-making is characterized by heightened risk, competitive awareness [49, 50], and assertiveness. Conversely, female executives tend to favor risk aversion [49, 51], collaborative management, and inclusivity in leadership [52]. Such behavioral tendencies suggest that the cognitive frameworks and operational modalities of male and female executives diverge markedly.

Drawing on these insights, this study asserts that gender plays a critical role in moderating the efficacy of ESG practices for enhancing corporate value. On the one hand, high self-confidence and a relatively aggressive approach are the prominent characteristics of male executives compared with female executives [53], which leads to different interpretations of the same ESG practices by male and female executives. Consequently, male executives may adopt a more aggressive approach to planning and deploying ESG practices in response to national and societal calls for environmental responsibility and governance, thereby influencing corporate value. On the other hand, compared with female executives, male executives, owing to their higher propensity for risk and willingness to engage in competition [54], are more likely to aim for increasing corporate market value in a shorter period by emulating benchmarks, enhancing corporate value by taking initiatives such as ESG practices, and having a higher tolerance for potential negative consequences of practicing ESG. In summary, in the process of improving corporate value through ESG practices, the distinct characteristics of male executives may amplify their impact of ESG practices on corporate value. Hence, the following hypothesis is derived:

**Hypothesis 3 (H**_**3**_**):** The male executives positively moderate the relationship between ESG practices and corporate value.

**Fig 1:**
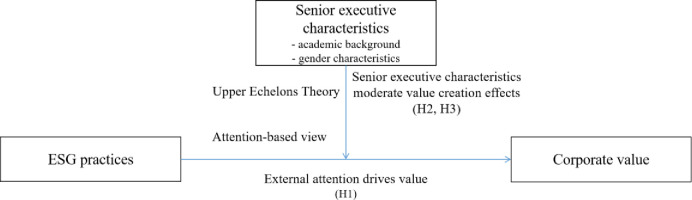
Conceptual framework illustrating the influence of ESG practices on corporate valuation and the moderating role of executive characteristics.

In summary, this study conceptualizes the mechanism by which ESG practices influence corporate value: Executive teams advance ESG initiatives driven by external focus, thereby impacting corporate valuation. Concurrently, executive characteristics significantly moderate the link between ESG practices and corporate value (Fig 1).

## Research design and methodology

### Data collection and sample selection

For this study, a longitudinal dataset comprising A-share listed corporations in China from 2009 to 2022 was developed. The following exclusion criteria were applied to refine the sample for analytical robustness:

Firms under special treatment (ST) or *ST owing to financial distress or irregularities were removed to maintain the standardization of financial indicators. Consistent with the established research protocols, the finance, insurance, and real estate sectors were excluded because of their unique financial structures. Instances with incomplete data pertinent to the study were discarded.

After these exclusions, we obtained a robust dataset comprising 35,449 firm-year observations. The primary data source for this study was the CSMAR database, which was supplemented with manual curation for accuracy. The CSMAR database is an authoritative database in the field of economics in China. It covers a wide range of research areas, including stock markets, companies, funds, bonds, derivatives markets, economics, industries, money markets, overseas markets, sectors, market information, special topics, technology finance, commodity markets, banking, and personnel tax collection. It currently comprises 140 databases. To mitigate the influence of outliers on the statistical analyses, a winsorization technique was employed at the 1% level for all continuous variables.

#### Variable definitions

*Independent variable*: *ESG practice (ESG)*. The independent variable was operationalized using the Huazheng ESG rating, categorized across nine levels, from AAA to C. These ratings were numerically coded from 1 to 9, ascending with the rating stature.

*Dependent variable*: *Enterprise value (value)*. The dependent variable, enterprise value, is the natural logarithm of market capitalization (market value A). This valuation encompasses the current market value of shares A and B, calculated at the current closing price.

*Moderating variables*: *Academic background (academic_c) and gender (sex_c)*. Academic background and gender are pivotal in examining the influence of executive characteristics. The former is attributed to chairs with a scholarly footprint: those who have engaged with higher education institutions, research bodies, or scholarly societies. Gender assessment was binary, identifying chairs as male (1) or otherwise (0).

*Control variables*. Control variables were incorporated based on the extant literature, accounting for factors known to influence enterprise value. These include enterprise age (age), leverage ratio (lev), return on assets (roa), equity multiplier (em), cash flow operations ratio (cfo), top five shareholder’ concentration (shrcr5), company growth (growth), asset structure (as), gross profit margin (gp), and board size (board). Table 1 delineates the detailed operationalization of these variables, providing a comprehensive framework for the subsequent empirical analysis.

**Table 1:** Operational definitions and measurement of variables.

Variable type	Variable name	Symbol of variable	Definition and measure of variable
Dependent	Enterprise value	Value	Natural logarithm of market value of the enterprise
Independent	ESG practice	ESG	Rating score of the enterprise’s ESG practices
Moderating	Executive characteristics	academic_c sex_c	Presence of academic background Gender identification as male or female
Control	Enterprise age	age	Current year minus the year of establishment
	Leverage ratio	lev	Ratio of total liabilities to total assets at the end of the period
	Net rate of return on total assets	roa	Ratio of net profit to total assets at the end of the period
	Equity multiplier	em	Ratio of total assets to owner’s equity at the end of the period
	Cash flow situation	cfo	Ratio of net cash flow generated from operating activities to total assets at the end of the period
	Equity concentration	shrcr5	Sum of the shareholding ratios of the top five shareholders
	Corporate growth	growth	(Operating revenue of the current year minus revenue of the previous year)/Revenue of the previous year
	Asset structure	as	Ratio of current assets to total assets at the end of the period
	Profitability	gp	(Operating revenue-operating costs)/Operating revenue
	Board size	board	Number of board members

#### Model specification

To empirically investigate the proposed hypotheses, this section outlines the econometric models designed for this study.

#### Baseline regression model for H1

The following baseline regression model was used to assess the direct influence of ESG practices on corporate value:

$$Value=\delta_{0}+\delta_{1}ESG_{it}+\delta CVs_{it}+\sum Industryfe+\sum Yearfe+\varepsilon_{it}$$ (1)

In Model (1), *i* indicates firms and *t* indicates time. CVs represents the control variables included in this study. Industry and Year are the fixed effects for controlling systematic differences across industries and years, and *ϵ* is the random error term. Consistent with H_1_, we anticipate *δ*_1_ to be positive, indicating that higher ESG scores are associated with greater corporate value. The baseline model uses ordinary least squares for regression.

#### Moderation model for H_2_

To examine the moderating effect of executives’ academic background on the relationship between ESG practices and corporate value, Model (1) was extended by including the interaction term:

$$Value=\delta_{0}+\delta_{1}ESG_{it}+\delta_{2}academic\_c_{it}+\delta_{3}ESG_{it}\times academic\_c_{it}+\delta CVs_{it}+\sum Industryfe+\sum Yearfe+\varepsilon_{it}$$ (2)

The coefficient *δ*_3_ captures the moderating effect of academic background on the ESG–value relationship. A significant *δ*_3_ would indicate a directional moderating effect. Based on H_2_, *δ*_3_ is expected to be negative, suggesting that the academic background of senior executives weakens the positive impact of ESG practices on corporate value.

#### Moderation model for H_3_

Similarly, to test H_3_ on the moderating role of executives’ gender characteristics, the following model includes an interaction term between ESG practices and gender:

$$Value=\delta_{0}+\delta_{1}ESG_{it}+\delta_{2}sex\_c_{it}+\delta_{3}ESG_{it}\times sex\_c_{it}+\delta CVs_{it}+\sum Industryfe+\sum Yearfe+\varepsilon_{it}.$$ (3)

In this model, a significant *δ*_3_ indicates that gender characteristics significantly moderate the ESG–value relationship. According to H_3_, we expect *δ*_3_ to be positive, reflecting that male senior executives enhance the positive effects of ESG practices on corporate value. The coefficients *δ*_1_ and *δ*_3_ in Models (1), (2), and (3) are of particular interest as they quantify the primary effects of ESG practices on corporate value and interaction effects of executive characteristics, respectively.

#### Estimation issue handling

Regarding testing for stationarity in panel data handling, generally speaking, panel data can be divided into macro panel data (with fewer entities N and longer time series T at the national level) and micro panel data (with more entities N but shorter time T at the level of firms or households). The stationarity of the panel data is important for panel data with several periods. Baltagi [55] suggests that, for macro panel data, data stationarity has a significant impact on the conclusions of the research when the time series is longer, involving unit root tests and cointegration analysis accordingly. However, in micro-panel data, especially when the number of observation periods T is short (2 years < T < 20 years), non-stationarity does not need consideration. Therefore, the stationarity of the micro-panel data used in this study was not tested.

## Empirical analysis and interpretations

### Descriptive statistics and correlation analysis

#### Descriptive statistics

Table 2 provides a comprehensive overview of the variables under study. The variation in corporate value across the sample, with a mean of 22.77 and substantial range, reflects differentiated market evaluations, possibly influenced by the extent of ESG practices, which themselves show variability with a mean score of 4.08. This variability in ESG scores suggests a spectrum of commitment to sustainability practices, hinting at the underlying strategic orientations toward corporate governance.

**Table 2:** Descriptive statistics.

	Count	Mean	SD	Min	p50	Max
Value	35449	22.771	1.123	20.284	22.609	26.586
ESG	35449	4.082	1.056	1.000	4.000	7.000
age	35449	18.091	5.913	2.000	18.000	36.000
lev	35449	0.421	0.202	0.033	0.414	0.924
roa	35449	0.035	0.069	-0.519	0.037	0.229
em	35449	2.061	1.183	1.033	1.703	9.635
cfo	35449	0.048	0.068	-0.174	0.047	0.291
shrcr5	35449	52.898	15.385	17.272	52.841	89.108
growth	35449	0.162	0.384	-0.653	0.104	3.324
as	35449	0.561	0.198	0.077	0.578	0.950
gp	35449	0.284	0.178	-0.075	0.249	0.870
board	35449	2.280	0.252	1.609	2.303	2.996
E	31059	1.880	1.166	1.000	1.000	9.000
S	31059	4.277	1.936	1.000	4.000	9.000
G	31059	5.371	1.424	1.000	6.000	9.000

Control variables such as the leverage ratio (lev) and return on assets (roa) offer additional context, with their means indicating a typical financial structure that can affect or be impacted by ESG practices. These figures, when read in conjunction with the asset structure (as) and profitability (gp), suggest the financial health and strategic priorities of enterprises, potentially impacting their valuation.

#### Correlation analysis

The analysis indicates an average variance inflation factor of 1.59, suggesting minimal collinearity among the variables. The correlation matrix in Table 3 represents the pairwise correlations of the variables. The positive and statistically significant correlation coefficient between corporate value and ESG practices (r = 0.17, p < 0.01) provides preliminary evidence in favor of H_1_, which posits that ESG practices are beneficial to corporate value. This initial finding is congruent with the thematic premise that ESG practices are value enhancing, although further analysis through multiple regressions is warranted to control for other potential influences.

**Table 3:** Correlation analysis summary.

Variable	Value	ESG	Age	Lev	Roa	Em	Cfo	Shrcr5	Growth	As	Gp	Board
value	1.000											
ESG	0.171***	1.000										
age	0.160***	-0.049***	1.000									
lev	0.386***	-0.110***	0.120***	1.000								
roa	0.105***	0.230***	-0.092***	-0.344***	1.000							
em	0.294***	-0.130***	0.098***	0.824***	-0.326***	1.000						
cfo	0.154***	0.104***	0.017***	-0.147***	0.396***	-0.148***	1.000					
shrcr5	0.114***	0.133***	-0.165***	-0.080***	0.210***	-0.064***	0.135***	1.000				
growth	0.081***	0.009*	-0.076***	0.023***	0.245***	-0.001	0.034***	0.060***	1.000			
as	-0.217***	0.060***	-0.113***	-0.152***	0.124***	-0.081***	-0.151***	0.016***	0.038***	1.000		
gp	-0.061***	0.092***	-0.070***	-0.440***	0.364***	-0.343***	0.250***	0.076***	0.069***	0.042***	1.000	
board	0.232***	-0.034***	0.084***	0.165***	-0.067***	0.138***	0.008	-0.007	-0.001	-0.134***	-0.079***	1.000

Robust heteroskedasticity-corrected t-values are presented in parentheses.

* p < 0 .10

** p < 0.05

*** p < 0.01.

## Regression analysis: Core findings

### Baseline regression results

Table 4 reports the test results of Model (1), where the dependent variable is corporate value (value) and independent variable is ESG practices (ESG). To prevent other factors from interfering with the results, this study controlled for all variables such as corporate age (age) and included fixed effects. As shown in column (1), the estimated coefficient for ESG is 0.1946, which is significantly positive at the 1% level. These results support the hypothesized expectation that ESG practices are significantly positively correlated with corporate value. Additionally, considering the different impacts of ESG dimensions on corporate value, this study examines the effects of the three ESG subdimensions on corporate value separately, where E, S, and G represent the indicators of environmental, social responsibility, and corporate governance, respectively. The regression results, as shown in columns (2)–(4), indicate that the estimated coefficients for E, S, and G are significantly positive at the 1% level.

**Table 4:** Regression analysis examining ESG practices and corporate value.

	(1)	(2)	(3)	(4)
	value	value	value	value
ESG	0.1946***			
	(19.6854)			
E		0.1387***		
		(13.2474)		
S			0.0647***	
			(11.2425)	
G				0.1046***
				(13.9023)
age	0.0096***	0.0074***	0.0089***	0.0073***
	(3.7188)	(2.6549)	(3.1160)	(2.6226)
lev	2.5273***	2.3359***	2.4122***	2.6543***
	(26.9209)	(23.5959)	(23.7921)	(25.6522)
roa	3.3339***	3.7191***	3.6756***	3.5042***
	(24.3727)	(25.2085)	(24.6871)	(23.8787)
em	-0.0015	0.0075	0.0031	-0.0089
	(-0.1007)	(0.4621)	(0.1907)	(-0.5542)
cfo	1.2042***	1.2609***	1.3497***	1.2901***
	(10.1644)	(9.6965)	(10.3319)	(9.9507)
shrcr5	0.0058***	0.0072***	0.0074***	0.0063***
	(6.4909)	(7.6297)	(7.7627)	(6.7632)
growth	0.0410***	0.0111	-0.0075	0.0091
	(2.8552)	(0.7534)	(-0.5071)	(0.6218)
as	-0.8700***	-0.8031***	-0.8580***	-0.8184***
	(-12.0011)	(-10.5332)	(-11.1023)	(-10.6961)
gp	0.2571***	0.2754***	0.1702**	0.1999**
	(3.3499)	(3.3249)	(2.0767)	(2.4265)
board	0.6458***	0.5954***	0.6153***	0.6470***
	(15.7317)	(13.7432)	(14.1649)	(15.0226)
_cons	18.7613***	19.2701***	19.4113***	18.7031***
	(115.4006)	(118.8010)	(120.2600)	(106.2493)
Industry	Yes	Yes	Yes	Yes
Year	Yes	Yes	Yes	Yes
N	35449	31059	31059	31059
adj. *R*^2^	0.397	0.392	0.383	0.387

Values in parentheses are t-statistics.

*** p < 0.01.

These findings suggest that companies with good ESG practices have higher corporate values. The executive team’s attention to the external environment can not only gain recognition from multiple stakeholders to enhance performance and promote corporate value but also improve environmental protection, social responsibility, and corporate governance, allowing companies to identify opportunities and enhance organizational performance. Moreover, all three ESG subcategories can promote corporate value, with the score for E having the most significant impact on corporate value and score for S having the least significant impact. This might be because, under the “dual carbon” context, environmental protection plays a more prominent role in enhancing corporate value. Thus, environmental protection has a significant impact on corporate value. The descriptive statistics of the remaining control variables are generally consistent with existing research.

### Examination of endogeneity test and robustness checks

#### Lag test for reverse causation

Endogeneity, particularly reverse causality in which high corporate value can lead to improved ESG practices, was addressed using a lag test. By analyzing ESG practices lagged by one period (ESG1), the estimations continue to show a significant positive effect (p < 0.01) on corporate value, as detailed in column (1) of Table 5. This supports the notion that ESG practices precede and contribute to changes in corporate value. The robustness of these results is further verified by lagging all control variables by one period, with the outcomes remaining consistently positive and significant.

**Table 5:** Robustness checks.

	(1)	(2)	(3)	(4)	(5)	(6)
	value	value	value	value2	value	value
ESG	0.1859***	1.9981***	0.2071***	0.1890***	0.1780***	0.1940***
	(18.3907)	(4.0088)	(36.2680)	(19.7471)	(33.3170)	(42.0649)
age	0.0063**	0.0172***	0.0092***	0.0053**	0.0091***	0.0098***
	(2.2641)	(2.9308)	(7.7543)	(2.0957)	(7.8932)	(10.4092)
lev	2.4778***	1.6635***	2.6150***	2.2321***	2.4203***	2.5219***
	(22.7941)	(5.7039)	(41.8627)	(24.5156)	(47.3345)	(57.4757)
roa	3.5601***	-2.6835	4.6467***	3.3326***	3.1633***	3.3319***
	(22.4143)	(-1.6355)	(34.9264)	(24.6324)	(30.4857)	(38.0960)
em	0.0061	0.2044***	0.0714***	0.0130	0.0145*	-0.0015
	(0.3244)	(3.6920)	(5.8960)	(0.9065)	(1.7205)	(-0.2135)
cfo	1.3118***	1.0385***	1.0526***	1.1246***	1.0592***	1.2261***
	(9.9313)	(3.1159)	(10.4812)	(9.7866)	(11.8850)	(15.6916)
shrcr5	0.0081***	-0.0117**	0.0050***	0.0102***	0.0072***	0.0057***
	(8.7474)	(-2.0463)	(12.4833)	(11.8680)	(19.0813)	(17.7755)
growth	0.0534***	0.3332***	0.0108	0.1159***	-0.0104	0.0405***
	(3.4768)	(3.5553)	(0.5905)	(8.1378)	(-0.7294)	(3.1512)
as	-0.7789***	-0.7643***	-0.9753***	-0.7169***	-0.8160***	-0.8883***
	(-10.1521)	(-4.4232)	(-27.4985)	(-10.2526)	(-25.7410)	(-32.1326)
gp	0.2507***	0.1951	0.2873***	0.4192***	0.1745***	0.2594***
	(3.0370)	(1.5079)	(7.1131)	(5.7114)	(4.6038)	(8.1550)
board	0.5911***	0.4966***	0.6904***	0.5950***	0.6098***	0.6507***
	(13.6036)	(5.9362)	(28.7130)	(14.9472)	(27.2220)	(34.1962)
_cons	18.8981***	14.5305***	18.6803***	18.9084***	18.8316***	18.7600***
	(111.5407)	(10.6379)	(175.7980)	(118.5206)	(235.0762)	(262.9351)
Industry	Yes	Yes	Yes	Yes	Yes	Yes
Year	Yes	Yes	Yes	Yes	Yes	Yes
N	30002	10698	24012	35449	23901	35276
adj. *R*^2^	0.395	.	0.420	0.394	0.414	0.398
F	.		363.3736	.	368.2576	476.9569

*** p < 0.01.

#### Instrumental variable approach

This study employs an instrumental variable method using the air quality index (PM2.5) of the firm’s location as an instrument to perform a two-stage least squares estimation. Superior air quality may reflect heightened societal ethical standards and environmental consciousness, which encourages companies to actively engage in ESG practices. Moreover, the regional air quality does not directly influence firm value, rendering the selected instrument exogenous. The results, displayed in column (2) of Table 5, align with previous findings and affirm the robustness of the study’s results.

#### Propensity score matching method

Propensity score matching was employed to address the potential sample selection bias. The process involved creating experimental and control groups based on ESG practice scores; conducting logit regression with firm age, industry, and year dummies and calculating propensity scores for matching. The matched-sample regression demonstrates that ESG practices have a significantly positive effect on corporate value at the 1% level (column (3), Table 5) confirming the stability of the results.

#### Alternative variable measurement method

To present a more comprehensive description of corporate value, this study introduced the natural logarithm of market capitalization B (value2) as an alternate variable. The formula for market capitalization B incorporates both A and B shares’ current values at the current closing price, adjusted for foreign capital in B shares. When replacing the original corporate value variable with value 2, the estimated coefficients of ESG practices (ESG) remain significantly positive at the 1% level (column (4), Table 5).

#### Subsample regression analysis

In response to external shocks, such as the COVID-19 pandemic and industry-specific practices, a subsample regression is used to test the robustness of the results. This research excludes data from 2020 to 2022 and samples from industries heavily skewed toward social responsibility, such as public facility management. This targeted analysis, detailed in columns (5) and (6) of Table 5, confirms that excluding these subsamples does not affect the overall pattern of results, indicating that the positive correlation between ESG practices and corporate value is not driven by these specific factors or time periods.

### ‎‎Exploring moderation effects: Academic background and gender

This study investigates the moderating effects of senior executives’ academic backgrounds and gender characteristics on the relationship between ESG practices and corporate value (Table 6). The analysis employed corporate value as the dependent variable, with the interaction terms between ESG practices and senior executives’ academic backgrounds or gender characteristics as the independent variables.

**Table 6:** Moderating effects of senior executives’ academic background and sex characteristics on ESG and corporate value.

Variable	Academic background (1)	Academic background (2)	Gender characteristics (3)	Gender characteristics (4)
ESG	0.2135*** (15.5381)	0.1981*** (17.6804)	0.0946** (2.3572)	0.0898*** (2.5857)
academic_c	0.0781 (0.8994)	0.1904*** (2.6588)		
ESGxacademic_c	-0.0459** (-2.0467)	-0.0515*** (-2.8050)		
sex_c			-0.2592* (-1.7515)	-0.2769** (-2.1307)
ESGxsex_c			0.1077*** (2.6296)	0.0972*** (2.7487)
Marginal moderating effect				
25% quantile	-0.0807*** (-5.3882)	-0.0747*** (-5.9669)	0.0466* (1.7213)	0.0762*** (3.8490)
50% quantile	-0.0546*** (-3.7461)	-0.0647*** (-5.2118)	0.1333*** (4.7251)	0.1088*** (3.2629)
75% quantile	-0.0901*** (-4.6583)	-0.0462*** (-2.9047)	0.1119*** (3.1525)	0.1163*** (3.6578)
age		0.0087*** (3.1866)		-0.2769** (-2.1307)
lev		2.4621*** (24.9943)		0.0972*** (2.7487)
roa		3.2938*** (23.1087)		0.0088*** (3.2107)
em		0.0078 (0.4940)		2.4623*** (24.9537)
cfo		1.2708*** (10.0522)		3.2976*** (23.2051)
shrcr5		0.0064*** (6.9345)		0.0070 (0.4451)
growth		0.0194 (1.3467)		1.2808*** (10.0993)
as		-0.8414*** (-11.2003)		0.0064*** (6.9825)
gp		0.2235*** (2.7600)		0.0173 (1.2028)
board		0.6301*** (14.9194)		-0.8465*** (-11.2978)
Constant	21.4841*** (173.8864)	18.7656*** (112.5469)	21.7716*** (119.6896)	19.0820*** (94.7068)
Fixed effect	Yes	Yes	Yes	Yes
Observation	31374	31374	31424	31424
Adjust R ^2^	0.162	0.403	0.161	0.404

*p < 0.10

**p < 0.05

***p < 0.01. ‎

Columns (1) and (2) of Table 6 show that the positive influence of ESG practices on corporate value persists even under the moderating effect of senior executives’ academic background. The interaction term (ESGxacademic_c) is negatively significant at the 1% level, suggesting that senior executives’ academic backgrounds may introduce a critical perspective that slightly mitigates the impact of ESG practices on corporate value, aligning with H_2_.

Columns (3) and (4) explore the moderating effects of gender characteristics. The interaction term (ESGxgender_c) is positively significant at the 1% level, indicating that gender characteristics of senior executives enhance the positive effects of ESG practices on corporate value. This finding supports H_3_ and implies that diverse gender perspectives on leadership may enhance the effectiveness of ESG practices.

Further examination of the marginal moderating effects in Table 10 reveals interesting dynamics across different quantiles. For the academic background interaction term (ESGXacademic_c), the regression coefficient decreases across quantiles (25%–75%), suggesting a more pronounced moderating effect in enterprises with lower corporate ESG practice scores. Conversely, for the gender characteristic interaction term (ESGXsex_c), the regression coefficient increases across quantiles, indicating a stronger moderating effect in enterprises with higher corporate ESG practice scores.

## Diverse impacts of ESG practices: Ownership, industry, and regional analysis

This section investigates the diverse impacts of ESG practices across enterprises, industries, and regions, considering the nature of enterprise ownership, high-tech industry status, and the regional institutional environment. It particularly focuses on the influence of these factors on the previously established relationship between ESG practices and corporate value, as well as the moderating effects of senior executives’ academic background and gender characteristics.

### Enterprise heterogeneity: Ownership nature

In Table 7, the analysis compares state-owned and non-state-owned enterprises in terms of the impact of ESG practices on corporate value. For state-owned enterprises, a significant positive correlation (coefficient: 0.2649, significant at the 1% level) exists, indicating a strong influence of ESG practices on corporate value than for non-state-owned enterprises (coefficient: 0.1207, significant at the 1% level). This suggests that state-owned enterprises are better positioned to leverage ESG practices to enhance their corporate value because of their close government ties and access to broader resources.

**Table 7:** Group test based on different ownership types: Impact of ESG on corporate value and moderating effects of executive characteristics.

	Baseline regression		Academic background		Gender characteristics	
	State-owned enterprises	Non-state-owned enterprises	State-owned enterprises	Non-state-owned enterprises	State-owned enterprises	Non-state-owned enterprises
ESG	0.2649*** (33.7700)	0.1207*** (22.3566)	0.2662*** (30.6283)	0.1167*** (17.3842)	0.0893** (2.1430)	0.0907*** (4.5023)
academic_c			0.3627*** (4.1215)	0.0627 (1.3670)		
ESGxacademic_c			-0.0646*** (-3.2634)	-0.0216** (-1.9848)		
sex_c					-0.6108*** (-3.3273)	-0.0261 (-0.3089)
ESGxsex_c					0.1709*** (4.0917)	0.0189 (0.9181)
age	0.0040** (2.3060)	0.0075*** (6.8698)	0.0045** (2.4490)	0.0065*** (5.6098)	0.0039** (2.1161)	0.0065*** (5.6428)
lev	2.2787*** (30.9656)	2.3971*** (44.2571)	2.1959*** (28.7167)	2.3418*** (41.1378)	2.2018*** (28.8169)	2.3398*** (41.0916)
roa	4.7769*** (25.7413)	2.9282*** (30.5284)	4.7468*** (24.7070)	2.8731*** (28.8802)	4.7428*** (24.7003)	2.8750*** (28.9246)
em	0.0145 (1.4242)	-0.0259** (-2.5599)	0.0258** (2.4296)	-0.0213** (-1.9857)	0.0227** (2.1318)	-0.0210** (-1.9643)
cfo	0.5343*** (3.9688)	1.3876*** (15.3290)	0.6247*** (4.5090)	1.4215*** (15.0172)	0.6155*** (4.4480)	1.4295*** (15.1002)
shrcr5	0.0194*** (35.6304)	-0.0031*** (-8.0021)	0.0196*** (35.0155)	-0.0025*** (-5.9450)	0.0195*** (34.7145)	-0.0024*** (-5.8025)
growth	-0.0489** (-2.1816)	0.1178*** (8.0020)	-0.0398* (-1.7330)	0.0893*** (5.9105)	-0.0391* (-1.7063)	0.0889*** (5.8866)
as	-0.6496*** (-14.8895)	-0.7419*** (-21.3191)	-0.6357*** (-14.1033)	-0.7230*** (-19.9053)	-0.6249*** (-13.8888)	-0.7278*** (-20.0392)
gp	0.0091 (0.1530)	0.4932*** (13.5169)	0.0155 (0.2515)	0.4474*** (11.5881)	0.0353 (0.5751)	0.4420*** (11.4727)
board	0.5396*** (16.4082)	0.3489*** (15.1950)	0.5485*** (16.1786)	0.3476*** (14.4499)	0.5536*** (16.3551)	0.3496*** (14.5424)
Constant	18.0737*** (158.8044)	20.1704*** (220.6192)	18.0369*** (153.6462)	20.1878*** (211.2506)	18.6764*** (86.9403)	20.2336*** (165.4187)
Fixed effects	Yes	Yes	Yes	Yes	Yes	Yes
Observations	-0.6108*** (-3.3273)	-0.0261 (-0.3089)	-0.6108*** (-3.3273)	-0.0261 (-0.3089)	-0.6108*** (-3.3273)	-0.0261 (-0.3089)
Adjust R ^2^	0.1709*** (4.0917)	0.0189 (0.9181)	0.1709*** (4.0917)	0.0189 (0.9181)	0.1709*** (4.0917)	0.0189 (0.9181)

***p < 0.01.‎

The table further explores the moderating roles of senior executives’ academic backgrounds and gender in these contexts. In state-owned enterprises, senior executives with academic backgrounds exhibit a significant negative moderating effect on ESG practices (coefficient: -0.0646, significant at the 1% level), reflecting a cautious approach. Conversely, the gender characteristics of senior executives, particularly in state-owned enterprises, show a positive moderating effect (coefficient: 0.1709, significant at the 1% level), aligning with the proactive and achievement-oriented nature often associated with male leadership styles.

Group coefficient tests further confirmed these findings and demonstrated significant differences at various levels of statistical significance. This underscores the variability in how ESG practices are perceived and implemented across different enterprise types, influenced by the inherent characteristics of the enterprises and backgrounds of their senior executives.

### Industry heterogeneity: High-tech vs. non-high tech

The relationship between ESG practices and corporate value varies significantly between high-tech and non-high-tech industries (Table 8). High-tech industries demonstrate a positive correlation coefficient of 0.1750 (significant at the 1% level) between ESG practices and corporate value. This finding indicates that ESG practices positively influence corporate value in these sectors. However, non-high-tech industries exhibit a stronger correlation, with a coefficient of 0.2247 (significant at the 1% level). This finding suggests that ESG practices have a more substantial impact on corporate value in these industries because of their unique markets and operational dynamics.

**Table 8:** Industry-based variation in ESG influence on corporate value.

	Baseline regression results		M = academic_c		M = sex_c	
	High-tech industries	Non-high-tech industries	High-tech industries	Non-high-tech industries	High-tech industries	Non-high-tech industries
ESG	0.1750*** (29.8477)	0.2247*** (30.9353)	0.1712*** (23.8305)	0.2346*** (27.9274)	0.1486*** (6.0041)	0.0268 (0.9306)
academic_c			0.1092** (2.0713)	0.2777*** (4.0302)		
ESGxacademic_c			-0.0327*** (-2.6545)	-0.0690*** (-4.2964)		
sex_c					0.0198 (0.1880)	-0.6269*** (-5.1875)
ESGxsex_c					0.0122 (0.4830)	0.1991*** (6.8370)
age	0.0168*** (14.1846)	0.0026* (1.6959)	0.0168*** (13.2134)	0.0004 (0.2287)	0.0169*** (13.3112)	0.0003 (0.1937)
lev	2.6565*** (46.4371)	2.2736*** (33.0838)	2.6057*** (43.2125)	2.1722*** (29.7250)	2.6065*** (43.2420)	2.1786*** (29.8872)
roa	3.1324*** (29.4393)	3.7165*** (24.9116)	3.0669*** (27.4473)	3.7316*** (23.7702)	3.0692*** (27.5004)	3.7223*** (23.7655)
em	-0.0305*** (-3.0070)	0.0312*** (3.1369)	-0.0253** (-2.3644)	0.0469*** (4.3270)	-0.0255** (-2.3881)	0.0438*** (4.0561)
cfo	1.4147*** (14.1985)	0.8896*** (7.2734)	1.5054*** (14.2567)	0.9370*** (7.3866)	1.5136*** (14.3506)	0.9483*** (7.4975)
shrcr5	0.0014*** (3.4708)	0.0117*** (22.8693)	0.0021*** (4.8114)	0.0121*** (22.5723)	0.0022*** (4.9374)	0.0120*** (22.5152)
growth	0.0905*** (5.4115)	-0.0316 (-1.6148)	0.0621*** (3.5743)	-0.0408** (-2.0350)	0.0614*** (3.5394)	-0.0442** (-2.2138)
as	-0.9131*** (-23.9694)	-0.7714*** (-18.3195)	-0.8845*** (-22.0472)	-0.7552*** (-17.1337)	-0.8874*** (-22.1337)	-0.7630*** (-17.3567)
gp	0.4192*** (10.4642)	0.0107 (0.2019)	0.3933*** (9.1160)	-0.0159 (-0.2892)	0.3856*** (8.9879)	-0.0175 (-0.3189)
board	0.6321*** (26.3848)	0.6033*** (19.8528)	0.6215*** (24.5210)	0.5808*** (18.2146)	0.6238*** (24.6431)	0.5836*** (18.3850)
Constant	19.0998*** (86.3765)	18.6673*** (186.8387)	19.1081*** (86.6071)	18.6920*** (178.1223)	19.1301*** (79.2510)	19.3576*** (125.4663)
Fixed effects	Yes	Yes	Yes	Yes	Yes	Yes
Observations	21651	13797	19005	12369	19038	12386
Adjust R ^2^	0.374	0.441	0.382	0.446	0.382	0.448

***p < 0.0.‎

The study also examined the moderating effects of senior executives’ academic backgrounds on this relationship. In high-tech industries, the interaction of executives’ academic backgrounds with ESG practices yields a coefficient of -0.0327 (significant at the 1% level), whereas in non-high-tech industries, it is -0.0690 (significant at the 1% level). This finding implies that the academic backgrounds of executives have a greater dampening effect on the influence of ESG practices in non-high-tech industries.

Furthermore, the analysis considers the moderating role of gender characteristics of senior executives. In high-tech industries, the interaction between these characteristics and ESG practices is 0.122; however, this is statistically non-significant. By contrast, in non-high-tech industries, the coefficient is 0.1991 (significant at the 1% level), indicating a more substantial effect of gender characteristics on the ESG–corporate value relationship in these sectors.

The group tests in Table 8 confirm these trends, highlighting the significant differences in how ESG practices impact corporate value across various industries and role of executive characteristics in this dynamic.

### Regional heterogeneity: Institutional environment variability

This study explores the impact of ESG practices on corporate value in diverse regional institutional environments, considering the influence of senior executives’ academic backgrounds and gender (Table 9). The analysis uses the marketization index as a proxy for the institutional environment with data spanning 2008 to 2021.

**Table 9:** Regional variability in ESG impact on corporate value.

	Baseline regression results		M = academic_c		M = sex_c	
	Better institutional environment	Challenging institutional environment	Better institutional environment	Challenging institutional environment	Better institutional environment	Challenging institutional environment
ESG	0.1545*** (22.8051)	0.2246*** (36.1263)	0.1652*** (20.5608)	0.2242*** (29.9040)	0.0928*** (3.5383)	0.0793*** (2.9309)
academic_c			0.1033* (1.7175)	0.2579*** (4.3819)		
ESGxacademic_c			-0.0341** (-2.4352)	-0.0646*** (-4.7020)		
sex_c					-0.1317 (-1.1831)	-0.4319*** (-3.7808)
ESGxsex_c					0.0639** (2.3884)	0.1327*** (4.8394)
age	0.0064*** (4.6135)	0.0119*** (9.3905)	0.0063*** (4.5131)	0.0108*** (7.4601)	0.0064*** (4.6156)	0.0109*** (7.5216)
lev	2.3206*** (33.4836)	2.6221*** (46.1702)	2.3271*** (33.5061)	2.5240*** (40.2274)	2.3199*** (33.4617)	2.5262*** (40.2874)
roa	3.3293*** (26.0348)	3.2813*** (27.7383)	3.3460*** (26.0170)	3.1660*** (24.4026)	3.3638*** (26.1898)	3.1590*** (24.3942)
em	0.0331*** (2.6675)	-0.0162* (-1.8748)	0.0329*** (2.6399)	-0.0050 (-0.5179)	0.0335*** (2.6985)	-0.0065 (-0.6796)
cfo	1.1733*** (10.1762)	1.3048*** (12.4326)	1.1621*** (10.0612)	1.4864*** (12.9176)	1.1699*** (10.1364)	1.5001*** (13.0530)
shrcr5	0.0036*** (7.3736)	0.0074*** (17.3378)	0.0036*** (7.3230)	0.0090*** (19.3056)	0.0036*** (7.3278)	0.0091*** (19.4787)
growth	0.0083 (0.4172)	0.0650*** (3.8982)	0.0085 (0.4269)	0.0303* (1.7201)	0.0060 (0.3035)	0.0291* (1.6561)
as	-0.8568*** (-20.5061)	-0.8790*** (-24.0200)	-0.8572*** (-20.4724)	-0.8194*** (-20.5508)	-0.8604*** (-20.5696)	-0.8257*** (-20.7548)
gp	0.1637*** (3.2625)	0.3213*** (7.8906)	0.1739*** (3.4559)	0.2776*** (6.1335)	0.1675*** (3.3379)	0.2806*** (6.2192)
board	0.6039*** (20.5937)	0.6415*** (25.8346)	0.5987*** (20.3707)	0.6066*** (22.2165)	0.6017*** (20.5142)	0.6098*** (22.3852)
Constant	19.2782*** (154.0490)	18.4462*** (202.6265)	19.2586*** (152.1287)	18.4564*** (189.1951)	19.4131*** (118.2843)	18.9339*** (130.9478)
Fixed effects	Yes	Yes	Yes	Yes	Yes	Yes
Observations	14745	20704	14708	16666	14716	16708
Adjust R ^2^	0.387	0.407	0.388	0.420	0.388	0.420

* p < 0.10

** p < 0.05

*** p < 0.01. ‎

The findings indicate that ESG practices enhance corporate value in both favorable and challenging institutional settings. The positive impact of ESG practices on corporate value is more pronounced in regions with less developed institutional environments. This could be because these enterprises face greater market challenges and recognize the benefits of ESG practices for value enhancement.

Senior executives with academic backgrounds have consistent, significant, and negative moderating effects on the relationship between ESG practices and corporate value across various institutional environments. Their comprehensive understanding of ESG, shaped by their academic and professional experience, makes them more cautious and thorough in implementing ESG strategies.

Furthermore, male senior executives exhibit a significant positive moderating effect on this relationship in both institutional contexts, with a stronger effect in less favorable environments. This suggests that in regions with limited institutional support, male executives may be more driven to leverage ESG practices to enhance corporate value.

## Pathways from ESG practices to corporate value: Mediation mechanisms

This section examines the pathways through which ESG practices influence corporate value. This suggests that ESG practices, when strategically integrated into corporate agendas, can potentially increase corporate reputation and augment government innovation subsidies. These improvements are hypothesized to attract attention in the capital and public markets, thereby fostering an increase in corporate value.

For the empirical analysis, this study employs factor analysis on a range of variables, including operating income and market valuation, to derive a composite corporate reputation score. Government innovation subsidies are deduced from the non-operating income section of financial statements. We used an SEM to confirm this pathway. The regression results indicate that the coefficient estimates for ESG on reputation and subsidies are significantly positive, demonstrating that ESG practices promote corporate reputation and government innovation subsidies (Table 10). The coefficient estimate of ESG on value remains significant, indicating the effective mediation of corporate reputation and government innovation subsidies. Table 11 employs standardized coefficients to report the direct, indirect, and total effects of the two variables. The direct, indirect, and total effects of ESG practices (ESG) is 0.0009, 0.2038, and 0.2047 (significant at the 1% level), respectively. Corporate reputation, having no indirect effect, has a direct effect and total effect of 0.2303. The use of standardized coefficients allows for the comparability of effect sizes. The direct, indirect, and total effects of government innovation subsidies are similar. This finding indicates that corporate reputation partially mediates the relationship between ESG practices and corporate value, with similar test results for government innovation subsidies. Therefore, ESG practices affect corporate value by influencing corporate reputation and government innovation subsidies, meaning the results support the pathway “ESG practices → Corporate Reputation/Government Innovation Subsidies → Corporate Value”.

**Table 10:** Structural equation modeling estimation.

	(1)	(2)	(3)	(4)
	Corporate Reputation		Government Innovation Subsidies	
	reputation	value	subsidy	value
ESG	0.3214***	0.0008	0 .0327***	0 .1084***
Reputation		0.5882***		
Subsidy				0 .1718***
Observations				
Adjust R ^2^				

* p < 0.10

** p < 0.05

*** p < 0.01. ‎

**Table 11:** Direct effect, indirect effect and total effect.

Impact Path	(1)	(2)	(4)
	Direct Effect	Indirect Effect	Total Effect
ESG > Reputation	0.8846***		0.8846***
Reputation> Value	0 .2303***		0 .2303***
ESG > Value	0.0009***	0.2038***	0.2047***
ESG > Subsidy	0.5652***		0.5652***
Subsidy > Value	0.0098***		0.0098***
ESG > Value	0.1074***	0.0055***	0.1130***

* p < 0.10

** p < 0.05

*** p < 0.01. ‎

## Discussion

As socioeconomic development faces complex external environmental challenges, China has emphasized harmonious relationships between humans and society and humans and nature. Therefore, companies’ ESG performance has received widespread attention from all sectors of society. This study empirically investigated the impact of ESG practices on corporate value and its mechanisms for listed A-share companies from 2009 to 2022. The main findings are discussed below.

First, this study explores the impact of ESG practices on corporate value through theoretical analysis and empirical testing. After a series of robust analyses, it finds the impact of ESG practices on corporate value statistically significant. This indicates that ESG practices have become necessary for the high-quality development of enterprises. With the goal of reaching a carbon peak and carbon neutrality proposed by China attracting attention both domestically and internationally, Chinese enterprises must pay attention to ESG practices and actively participate in environmental, social welfare, and corporate governance activities, thereby helping in achieving the goal of enhanced corporate value.

Second, the relationship between ESG practices and corporate value is moderated by executive characteristics. Specifically, academic background of executives has a negative moderating relationship between ESG practices and corporate value, whereas male executives have a positive moderating relationship. Under the current drive toward the “dual carbon” goals in our country, enterprises need to actively optimize the executive team structure based on the characteristics of executive traits, establish a more rational talent echelon, and continuously enhance corporate value.

Third, the impact of ESG practices on corporate value and moderating role of executive academic and gender characteristics vary among companies. It is more pronounced for state-owned enterprises, non-high-tech industries, and those in poor institutional environments. The negative moderating relationship between senior executives’ academic backgrounds and the relationship between ESG practices and corporate value is stronger in state-owned enterprises and non-high-tech industries. Moreover, the positive moderating relationship between male executives and the relationship between ESG practices and corporate value is strengthened in state-owned enterprises, non-high-tech industries, and those in poorer institutional environments.

Finally, corporate reputation and increased government innovation subsidies mediated the impact of ESG practices on corporate value. China’s economy has transitioned from a high-speed growth phase to a high-quality development stage, in which enterprises serve as crucial micro-foundations for promoting high-quality economic development. In the process of value creation, enterprises should focus on promoting sustainable development by enhancing their corporate reputation. Simultaneously, ESG practices should be integrated with corporate innovation, optimizing resource allocation, and reducing corporate costs by better use of government innovation subsidies, thereby continuously improving the overall value of the enterprise.

Although this study offers comprehensive insights, it has some limitations. Future research should examine how different institutional environments, tenure lengths, and senior executive positions influence the relationship between ESG practices and corporate value. Additionally, expanding the dataset beyond the Sino-Security database and incorporating industry-specific micro-survey studies can provide more granular insights into the impact of senior executives’ academic backgrounds on corporate ESG practices and value creation.

## Conclusions and implications

### Conclusions

Through theoretical analysis and empirical testing, this study explores the impact of ESG practices on corporate value and its mechanisms. The following conclusions are drawn: First, the better the ESG practices, the higher the corporate value. Second, executive characteristics moderate the relationship between ESG practices and corporate value. Third, the promotion of corporate value by ESG practices is implemented by enhancing corporate reputation and increasing government innovation subsidies. Finally, the aforementioned impact relationships and moderating effects differ across different ownership types, industry characteristics, and institutional environments. This study provides micro-level evidence supporting the positive economic consequences of ESG practices and offers insights for companies and investors to value ESG practices and government departments to improve ESG incentive policies.

### Managerial implications

These findings highlight the significant impact of ESG practices on corporate value and provide crucial insights for business leaders, investors, and policymakers. These results emphasize the importance of integrating ESG considerations into corporate strategies to enhance company value. Government regulators should enhance the disclosure requirements for ESG information and implement reward and punishment systems to promote transparency and accountability. This will guide investors toward a more sustainable investment philosophy and encourage companies to be more proactive in their ESG disclosures.

For businesses, this study suggests a shift in perspective on ESG practices. Rather than viewing them as resource-intensive, businesses should recognize their potential to enhance their corporate reputation and attract government innovation subsidies. Implementing a robust ESG framework and aligning it with performance management systems can promote long-term sustainability. Additionally, improving corporate governance and stakeholder relationships is crucial for fostering a business environment conducive to ESG practices.

This study also highlights the role of senior executives’ academic backgrounds and gender in moderating the relationship between ESG practices and corporate values. This insight can be instrumental in strategic human resource decisions and leveraging the unique roles of academically inclined senior executives in advancing corporate ESG agendas.

## Supporting information

S1 Dataset (ZIP)
